# DSD and Professionalism from a Multilateral View: Supplementing the Consensus Statement on the Basis of a Qualitative Survey

**DOI:** 10.1155/2012/185787

**Published:** 2012-07-09

**Authors:** Jürg C. Streuli, Birgit Köhler, Knut Werner-Rosen, Christine Mitchell

**Affiliations:** ^1^Institute of Biomedical Ethics, University of Zurich, Pestalozzistraße 24, 8032 Zurich, Switzerland; ^2^Department of Pediatric Endocrinology, University Children's Hospital, Charité, Humboldt University, 13353 Berlin, Germany; ^3^Office of Ethics, Children's Hospital Boston Division of Medical Ethics, and Harvard Medical School, Boston, MA 02115, USA

## Abstract

Treatment and support of a child with DSD calls for experience and expertise in diagnosis, surgical techniques, understanding of psychosocial issues, and recognizing and accepting the significance of individual values of children, families, and support groups. The range of what is considered “appropriate” care and treatment is still very broad and critics point at major gaps between ethical guidelines and current clinical practice. Based on a qualitative study with 27 members of multidisciplinary teams and support groups, we supplement the professional consensus statements and current ethical guidelines with 14 requirements from four different perspectives, to characterize more fully the responsible treatment and support of children and families affected by DSD. Overall, our findings highlight the importance of close collaborations between different experts and a shift from the often simplified dispute about genital surgeries to a more holistic perspective with a long-term management strategy, which should serve as a cornerstone not only for clinical practice but also for future research and evaluation studies.

## 1. Introduction

The care of a child with a disorder or difference of sex development (DSD) requires a long-term management strategy that should involve several health professionals who possess the ability to support such children and their families, providing care, protection, and participation in decisions [1]. Frontline primary and specialist health professionals must recognize such children's vulnerability, their needs, their rights, their evolving capacities and contextual aspects including their parents' fears, values, and beliefs. The well-being of children with DSD, their right of integrity, need for protection, prospects for autonomy, and capacity to participate in informed consent or assent, all, vary individually in accord with the child's development and history [2–4].

In recent years professionals have gained significant experience and expertise in diagnosis, surgical techniques, understanding of psychosocial issues, and recognizing and accepting the significance of individual values of children, families, and patient advocacy groups [1, 4–6]. In 2005, the Consensus Statement Conference pointed at major shortfalls in care, treatment, and support of patients with DSD, issued new standards of care, and highlighted the need for multidisciplinary teams involved in the long-term care of patients with DSD and their families [5, 6]. Accompanied by the transformative effect of efforts within the Lawson Wilkins Pediatric Endocrine Society (LWPES), the European Society for Paediatric Endocrinology (ESPE), and the German Network DSD/Intersex, communication and psychosocial support has especially improved [6–8]. However, recent publications still point at gaps between ethical guidelines and clinical practice [7–12]. Confidence in using precise etiology and defining objective outcomes may not be sufficient to determine the appropriate management of a heterogeneous group of children and young adults with DSD [13]. The range of what is considered “appropriate” care and treatment is still very broad and has only theoretically diminished. Although a consistent terminology and a multidisciplinary approach are useful, the specific skills needed to help growing children and their parents make decisions and cope with DSD are still not well delineated. 

We use here the term “professionalism” to refer to skills and related conduct, aims, and qualities that ultimately characterize expert and ethical healthcare providers [14, 15]. Professionalism can be seen as a normative and descriptive commitment to carrying out professional responsibilities, as an adherence to professional ethical principles, and as sensitivity to unique and diverse patients, including those with DSD [16]. We believe it is not sufficient to know simply about ethical principles and clinical recommendations. What is also needed is sensitivity regarding a variety of interactional and dilemma-specific scenarios in which different ways of addressing and resolving problems and avoiding harms can be developed and assessed with the aid of an ethical professional experienced in the care of patients with DSD and knowledgeable of the history and evolving standards of medical and psychosocial care [17]. Professionalism is not merely a collection of knowledge and ready-made skills; it also requires self-examination and awareness of one's values and attitudes about children, families [18], and sexuality, as well as defined procedures which can be evaluated and compared between different providers. Ethics, as a reflection on values, principles, and responsibilities focused on choosing the good and right action, is an essential aspect of professionalism [19]. 

The concept of “multilateralism” was earlierly associated with professionalism by the Committee on Bioethics of the American Academy of Pediatrics [16]. It is a common term in international relations pointing at the effort to incorporate different interests and abilities of various stakeholders on an egalitarian basis. The concept of shared decision making [20] and the ongoing changes in the care of families affected by DSD are closely related to a multilateral perspective. 

Although there is a growing body of literature concerning what professionalism in general means, there is little discussion, research, and education regarding the care of persons with specific problems like DSD [20, 21]. Therefore we asked about particular requirements and needs for an adequate care of children and families affected with DSD. For answering this question we analyzed interviews of members of multidisciplinary teams and supporting groups, focusing on their understanding of professionalism as well their underlying opinions, needs, and problems concerning DSD.

## 2. Methods

From 2009 to 2012 we conducted several projects studying attitudes and procedures in the context of DSD. Data were collected in one-on-one interviews (*N* = 20) and one focus group accompanied by a review of the literature. The semi structured interviews ranged from 35 minutes to 2 hours and contained at least one case example (parents of a newborn child with ambiguous genitals who are uncertain about what to do) as well as questions concerning relevant values, principles, and facts. All participants were either part of a multidisciplinary team (in total we studied 5 teams in Switzerland and Germany) or part of a support group (2 mothers, 1 patient, and 1 activist). In total, 27 persons were included. A list of professional background and current clinical experience (represented with the number of treated or supported DSD patients or families per year) is shown in Table 1. All interviews were transcribed verbatim. Transcripts were entered into a qualitative data analysis program (ATLAS.ti. 2011, Version 6.2. Berlin, Scientific Software Development). Interviews were conducted in German. Relevant and cited parts of the transcript were translated by an independent translator and double-checked by one of the author. 

## 3. Data Analysis

Investigators developed a preliminary framework of descriptive, normative, and linguistic information extracted from participants' statements, resulting in themes to outline the key points of professionalism concerning DSD. The analysis was based on a modified method of interpretative phenomenological analysis facilitated by software for content analysis (ATLAS.ti, 2011, version 6.2, Berlin, Scientific Software Development). The institutional review board of the Canton of Zurich approved the study.

## 4. Results

The content analysis revealed 14 abilities or skills, which we have grouped into four categories of professionalism listed in Table 1, which lists the frequency (in absolute numbers) of codified interview passages for themes passages for higher-order themes substantiated by selected samples from the interviews. 

## 5. Child-Oriented Professionalism

A main goal of pediatrics is to provide the opportunity for children to achieve their maximum potential for coping with current and future demands. The same can be stated for DSD as follows.

*Neonatologist: “We mainly talked about the parents' perspective. The most important, however, is the individual, the child. And if we have ethical questions, we always should take into account where and how this particular child will be in twenty years. That's the main point.” (ID21:89). *

Focusing on the long-term development of the child rather than just initiating a one-time treatment was seen by some of the interviewees as a central goal of DSD treatment and in contrast to the criticized and perhaps clichéd attitude of “just fixing the problem” by surgery [9, 20]. 

Professionals also have to acknowledge that a child's development, future well-being, and preferable outcome in quality of life can have different important aspects, which might be related to different backgrounds and disciplines.

*Pediatric surgeon: “[*⋯*] well that is actually quality of life, that one is not incontinent, that no urinary tract infection comes about, that it is separate, that the urine does not run backwards continuously or anyhow, that is quality of life, yes.” (ID16:4).*

*Resident in endocrinologist: “[Quality of life means] acceptance by fellow human beings, acceptance by any partner or friends, homosexual or as well heterosexual, that I think is of utmost importance [*⋯*].” (ID8:13). *

There are some important differences between fixing and supporting, but both should have in common an appreciation of their potential to harm the child. One of the major points raised by critics of medical treatment is the lack of sensitivity for therapeutically induced harm and the long history of applying surgical procedures without clear evidence of benefits for the child or the future adult (e.g., appearance-normalizing surgery in childhood).

*Pediatric Surgeon: “We enter this dilemma every time: What is the best [for the child]? [*⋯*] Each time we have to ask us anew, saying where the road is going*⋯* are we still allowed to do that operation, shall we continue this treatment or*⋯* I think this is most central. Moreover we should harm as little as possible, that's very important.” (ID16:1).*

*Mother: [What makes my child suffer, is] to constantly afflict the child with some checkups, to exhibit the child constantly at different doctors. Constantly to look at the genital [area], let others look at it, make photos of it, these are things which are not necessary from my point of view, because I think today there is enough material available, so it should not be necessary to mug every child. (ID23:7).*

According to both interviewees the principle of not harming (nonmaleficence) has three components: first, to do only what is really necessary, second to do it as harmlessly as possible, and third—if a certain intervention is necessary—to obtain consent and therewith to explain why it is important. Even common procedures such as physical examinations can deeply hurt the child's and the parents' feelings [22]. 

By focusing on a child-oriented professionalism health professionals and parents may apply their adult logic and ideas to a child's world. One of several potential burdens for a child with DSD, which was regularly mentioned as a rationale for cosmetic surgery, was the swimming class scenario, with potential exposure in changing rooms or bathing suits as a major problem. However, as the following sample shows, this should on the one hand be taken as a serious problem but on the other hand also critically questioned.

*Specialist in sexual medicine: “[the swimming class as a real problem for children with unoperated DSD] seems to be constructed (*⋯*). It has never been delivered to me like that and it seems to arise from adult logics—Therefore one has first of all to make plausible that the feeling of exclusion develops through the difference of genitals in a seven year old and that again has to be taken seriously. If a child feels excluded it is for me a comprehensible psychosocial emergency. One has to think about how to react on that, but that is for me by no means an indication for surgery, however, I would take it very seriously.” (ID13:10). *

## 6. Family-Oriented Professionalism

Childhood is a period of relative vulnerability and dependency [23] and relationships play a particularly important role.

*Interviewer: “Can you tell me something about the quality of life of the person that you imagined in her adult life, in her future?”*

*Child and adolescent psychiatrist: “Well actually [she's doing] very well, because she is well embedded in her family, because she has stability in herself, also with respect to her difference [*⋯*].” (ID14:9).*

*Neonatologist: “The most important is bonding [between the child and its parents]. [*⋯*] First there must be a bond, the rest is coming after” (ID22:8).*

For health professionals helping parents accept and bond with their child is a recognized priority, which requires the ability to model that behavior for parents as well as to integrate parents' own reactions, needs, and values. Parents are of essential importance for children and have not only the right but also the duty to make certain decisions for their children. However, neither the duty nor the right is absolute and may be guided (or, in rare cases, overridden) when a parent's decision is clearly and seriously harmful to their child. Professionals who attend to parents' reactions, values, and needs are better able to coordinate adequate support or interventions. Regardless of whether surgery would be an option when a family is not able to cope with the uncertainty of DSD or not, the decision should always be made in close collaboration with different experts who have the knowledge of alternative and complementary options such as day care, home health services, foster families, rehabilitation services, and communal psychosocial and intercultural support.

*Social Worker: “[*⋯*] to see the overall patient in his surroundings as well and this is also part of our concept, to support the family in a way that the patient gets as much help as possible and that he is not bothered due to additional difficulties [*⋯*], simply to see, which support structure fits, this is already the first step independent of the disease.” (ID18:1).*

*Endocrinologist: I had a child, where the parents said: I don't take this child like that. Well, then the child went to foster care, somehow, and two years later, they still don't get the point [with DSD] but at least they, as I think, do well with their child now. It is as a matter of fact just a difficult topic. But, however, I believe that parents must have an emotional bonding with their child, but at the same time not being absolutely fixed on a certain gender. Somehow this can be supported independent of each other. (ID3:6).*

A cornerstone of any successful treatment and support seems the ability of parents and their children to cope with different scenarios. Enabling parents and children to cope may not only involve professional intervention but also nonintervention by leaving enough room and time for emotions and reflection.

*Specialist in sexual medicine: “On the other hand it is about checking with the parents to see, how can they deal with the negative feelings, how can they reinterpret or see them differently [*⋯*]. It is also about offering the child gradually an ideal concept, less in the sense of, now it is two or three years old and this or that conversation should take place, but rather to stay in contact with the child and see what questions emerge, what confrontations appear.” (ID12:2).*

## 7. Shared-Decision-Oriented Professionalism

Many professionals emphasized in the interviews that a decision is not a one-time event nor does a decision fix something forever. Rather, a decision is part of a process, which must be made in close contact with all the decision makers.

Although decisions in early childhood must be made by proxies, that is, by parents on behalf of their child, it should be clear from the beginning how the child can and will be involved during its development. Some interviewees, therefore, supported the idea of postponing as many irreversible interventions as possible to an age when the child can be an active part of decision-making.

*Social worker: “[*⋯*] one needs to think long-term oriented, it is again about self-determination. [*⋯*] Therefore strengthening the parents so that the child can grow as it is, and once it becomes sexually mature, when it can start to think about it, be able to decide [about medically not indicated surgery].” (ID18:11).*

*Pediatric surgeon: “That's the key point itself; to say that only those operations should be done which are necessary according to current evidence.” (ID16:7).*

To distinguish between necessary and unnecessary interventions and to make a sound decision in general, professionals have the difficult task of distinguishing between facts and assumptions and informing parents and patients about the uncertainty associated with current data.

However, especially as knowledge and recommendations for medical and surgical treatments for DSD change, it is important to be aware not only of the values of the family, the growing child and the future adult but also of the health professional's own reactions and underlying values. This might not be an easy task, as a resident in endocrinology noted:

* “I do not know how it would feel if I had an own child with peculiar genitals, I think it would be very different then, and one would think a lot more about it. It just is like that, we do see so many patients with diverse dysplasias or dysfunctions, (*⋯*) maybe one numbs oneself to it, well, that could of course also be, that “professionalism” with quotation marks then has something to do with getting numb, hmm.” (ID8:1). *

In our interviews professionalism was often described as a professional distance from the problem, which helps to see questions and problems from a more comprehensive point of view, with a certain neutrality, emotional distance, and impersonality. Healthcare workers should be aware that this not only makes them feel distant from the problem but maybe also distant from the feelings and reactions of children and parents. The transition from being distant in a professional way to developing a form of numbness usually happens without awareness and therefore should be pro-actively avoided. 

By studying the interviews, it became clear that values of medical professionals are neither universal nor do they have to be identical with family or patient-specific values. Since the interpretation of empirical data on DSD is highly dependent on values, professionals should be especially careful not to pathologize or medicalize the child as a clinical problem—narrowly seen and labeled as, for example, an enzyme defect, an ambiguous genitalia baby, “a CAH child,” or a tumor risk, instead of “Sam” or “Chris” who has a certain problem or diagnosis and is getting ready to attend kindergarten.

*Specialist for sexual medicine: “[The expert] has to appreciate the pathologies and to make sure at the same time that those problems won't dominate the decision-making process. [Medicine] has to see that it has to de-pathologize the subject, also with respect to the consequences for the whole family.” (ID13:3).*

A therapy is not able to fix the uncertainty for certain; the ambivalence will prevail at least in certain situations (as shown in the next sample). Professionals should be able to support the parents and the child to handle those situations in a positive way.

*Interviewer: “Today it's called disorder of sex development.”*

*Father: “But it is NO disorder!”*

*Mother: “Well, actually it is.”*

*Father: “NO! I don't think it is a disorder.”*

*Mother: “Well, it is not a mistake neither.”*

*Father: “No.”*

*Mother: “Hmm, it's really difficult.” (ID23:12). *

## 8. Quality-Oriented Professionalism

Whether a surgeon is doing surgery on a child's genitals or a psychologist is talking with parents about their fears and needs, both are clinical interventions and both can be done in a more or less professional way. In the last 20 years certain errors in earlier treatment regimes have become well known and were mentioned by almost all interviewees. Moreover, according to the interviews, most professionals developed a critical distance to their own treatment regime:

*Pediatric Surgeon: “Well, the really critical statements [against genital surgery] are not coming from CAH-groups but from others. No doubt they are justified and it helped to raise our awareness and in that sense the discussion for me is positive, in a way that the clitoris should be saved, spared and [smiling] caught our full attention again. [*⋯*] [T]his even makes sense as a possibility and the ongoing development in this field will give us more knowledge.” (ID16:10).*

One strategy to improve the standard of care and one of the main points of almost all interviewees is the limitation of certain procedures to fewer “validated” clinics with necessary skills and resources:

*Resident in endocrinology: “I think that some surgeons simply overestimate their abilities and one therefore should really know very well who is able to do what, but I believe as well that many just do not like to transfer [their patient] to another center.” (ID9:11). *

*Surgeon: “I know also some urologists, who might do that [genital surgery] occasionally as a hobby, but yes, what is missing there in my opinion is the close collaboration with the endocrinologist and therefore, I do think that the pediatric endocrinologists have to centralize among each other, that they say, well in fact those special cases, with this problem they have to go in these two centers [...] because then of course, I'd say, a new trend will emerge, new standards, new leadership will become accepted.” (ID15:12).*

## 9. Discussion

The 2005 Consensus Statement summarized major changes in the care, treatment, and support of patients with DSD and pointed to the need for multidisciplinary teams. Subsequently we analyzed qualitative interviews of members and collaborators of such multidisciplinary teams, to understand and systematically summarize their opinions about the necessary qualifications of those caring for persons affected by DSD. 

Our findings led to a list of 14 abilities and skills within four categories of professionalism (child-oriented, family-oriented, shared-decision-oriented, and quality-oriented professionalism), which might help to educate and evaluate multidisciplinary health professionals implementing the care and management of children, with DSD as described in the consensus statement. The qualitative approach enabled an in-depth and nuanced study of those questions and an important supplement to existing guidelines as well to other quantitative and qualitative surveys [6, 9, 23, 24].

The results suggest that the high requirements of professionalism for those involved in the care and treatment of persons with DSD can best be realized in experienced multidisciplinary teams with an active culture of long-term multilateral interaction between parents, their growing children and various experts including supporting groups. The studied multidisciplinary teams showed an impressive broadness of knowledge and various skills essential for collaborations among those experts treating and supporting children or adults with DSD. Overall, we note a growing resistance to surgical gender assignment and sensitivity toward comprehensive and systemic support of the child and its family. These heterogeneous opinions suggest not only openness but also uncertainty among professionals about the right approach, which potentially passes to both parents and patients. As the highly debated question about whether and when to do surgery becomes less central, critical but constructive in-depth discussion about what constitutes professional treatment and support of persons and families affected by DSD is timely and needed. This is of particular importance, because treatment, research, and critics still often focus mainly on the consequences of surgery [9, 23, 25]. Our results, however, indicate that it might be of much greater importance to assure that parents and children are supported in a professional, multilateral, and sensitive way, embracing the aspects of professionalism identified from the interviews and summarized in Table 2. Whether there was a cosmetic operation or not may not be as critical to good outcomes for these patients and the future quality of their lives as how the person was supported and educated by parents, healthcare professionals, and supporting groups. As a consequence, this would mean that results trying to show parents or patients, who regret the fact that there had or had not been a cosmetic operation in childhood [23, 25, 26], are based on a somewhat misleading interpretations of the underlying problems, due to a narrow focus on surgical outcome. Of much more significance might be the question whether the overall support was done in a professional way as indicated here.

While we would not claim that every child and every adult with DSD who show a negative outcome, such as poor self-esteem [2, 12] or dissatisfaction with overall sex life [27], are exclusively the result of deficient adherence to professionalism, we are concerned that the professionalism of healthcare providers be directed effectively toward dealing with and diminishing negative outcomes. There is not yet, however, sufficient evidence to fully support this particular understanding of professionalism nor has there been an adequate discussion so far about the resources it would take to fulfill the very high demands of the presented interpretation of professionalism. Nevertheless we believe these data refer to key aspects of professionalism needed to improve care for persons with DSD. Professionalism contains a normative claim for responsibility and provides direction for new research projects. It also underlines the need for centralizing competencies to a few specialized centers with well-connected, experienced and up-to-date professionals, representing and realizing the culture of a holistic, multidisciplinary care.

The study was limited by a relatively small sample of participants in only two European countries. It is not sufficient to establish the consistency and generalizability of statements nor do we claim that the summary offered in Table 2 is a complete set of defining characteristics of the concept of professionalism in caring for persons with DSD. We have, of necessity, presented short samples of different statements, which permit only a limited perspective on the multifaceted and differentiated opinions of the interviewed professionals. 

With these limits in mind, we share the findings from our qualitative analysis of professionalism and DSD to glean preliminary insights into the current practice of multidisciplinary teams including their problems, needs, and skills. Overall, findings underline the importance of close collaborations between different experts and point to the responsibilities, which are related to the provision of care, support, and treatment of persons with DSD.

## 10. Conclusion

In view of the comprehensive and highly influential consensus statement, our results pointed at different enduring difficulties: first, a need for discussion about ensuring quality and education; second, a need for better collaboration and provision of various options complementary or alternatively to surgical and medical treatment; third, a need for greater skills related to an improved awareness of the impact of medicalized communication and problem-solving and fourth, strengthened abilities to educate parents and their children towards uncertainties in current medical treatments, giving them as much information and choice as possible for coping with current and future situations. In conclusion, the analysis reinforced the merit of a multidisciplinary team and made the often studied, discussed, and criticized genital surgery a secondary consideration. A nuanced understanding of professionalism concerning DSD embraces the ability of different disciplines, experts, and support groups to provide treatment and support of affected persons in a responsible, comprehensive but sensitive way as summarized here.

## Figures and Tables

**Table 1:** Participants and their background.

ID	Function	Current practice^∗^
1	Pediatric endocrinologist 1	A
2	Pediatric endocrinologist 2	B
3	Pediatric endocrinologist 3	B
4	Pediatric endocrinologist 4	D
5	Pediatric endocrinologist 5	D
6	Pediatric endocrinologist 6	C
7	Pediatric endocrinologist 7	A
8	Resident in endocrinology 1	C
9	Resident in endocrinology 2	C
10	Psychologist 1	B
11	Psychologist 2	D
12	Specialist in sexual medicine 1	C
13	Specialist in sexual medicine 2	C
14	Child and adolescent psychiatrist	C
15	Pediatric surgeon 1	C
16	Pediatric surgeon 2	B
17	Pediatric surgeon 3	C
18	Social worker	D
19	Study nurse	B
20	Gynecologist	C
21	Neonatologist	C
22	Members of support group 1 (*N* = 2, parents of a child with DSD)	D
23	Member of support group 2 (*N* = 1, mother of a child with DSD)	D
24	Members of support group 3 (*N* = 3, mother, activist and patient)	C

*Current experience represented in number of patients or families with DSD supported or treated per year: A: over 50, B: 15–50, C: 5–14, D: 1–4.

**Table 2:** Summary of resulting categories concerning professionalism on DSD.

Different views on professionalism	Themes	Quotations (*N*)
Child-oriented professionalism	Awareness of the importance of development	21
	Attention to a growing child's quality of life and physical as well as psychosocial integrity	10
	Ability to recognize therapeutically induced harm and to balance it with evidence-based benefits	13
	Compassion and empathy based on understanding a child's reaction and sensitivity regarding differences between children's and adult's perspectives	25

Family-oriented professionalism	Appreciate the importance of relationships	17
	Multilateral interactions between child, parents, professionals, and support groups	23
	Skills to identify and integrate parents' reactions, values, and needs and the ability to coordinate adequate support and interventions	37
	Ability to support parental empowerment, coping and resilience	23

Shared-decision-oriented professionalism	Development and ongoing adjustment of treatment plan in close collaboration with patient and parents and based on their assent/consent	14
	Awareness and communication of all available or missing evidence concerning treatment and outcome	26
	Awareness of one's own values, feelings, and influence	19
	Sensibility and avoidance of unnecessary medicalization	37

Quality-oriented professionalism	Ability to acknowledge, evaluate, communicate, and improve substandard quality	18
	Limiting certain interventions to centers and experts with necessary experience and skills	14

## Abbreviations

DSD: Disorders of sex development
ID: Number of identification of participants.
